# Demographic and Clinical Characteristics of Mild, Young and Early COPD: A Cross-Sectional Analysis of 5468 Patients

**DOI:** 10.3390/jcm13237380

**Published:** 2024-12-04

**Authors:** Cristina Aljama, Cristina Esquinas, Eduardo Loeb, Galo Granados, Alexa Nuñez, Ane Lopez-Gonzalez, Marc Miravitlles, Miriam Barrecheguren

**Affiliations:** 1 Department of Pneumology, Universitary Hospital Vall d’Hebron/Vall d’Hebron Institut de Recerca (VHIR), Vall d’Hebron Barcelona Hospital Campus, 08035 Barcelona, Spain; cris.aljama94@gmail.com (C.A.); crise4@hotmail.com (C.E.); galodavid.granados@vallhebron.cat (G.G.); alexagnd01@hotmail.com (A.N.); anelopez95@gmail.com (A.L.-G.); miriam.barrecheguren@vallhebron.cat (M.B.); 2 Department of Public Health, Mental Health and Maternal and Child Health Nursing, Faculty of Nursing, University of Barcelona (UB), 08007 Barcelona, Spain; 3 Department of Pneumology, Centro Médico Teknon, Grupo Quironsalud, 08023 Barcelona, Spain; eloebm@gmail.com; 4 CIBER de Enfermedades Respiratorias (CIBERES), 08035 Barcelona, Spain

**Keywords:** COPD, young, early, mild, prognosis

## Abstract

Early, mild and young COPD concepts are not clearly defined and are often used interchangeably to refer to the onset of the disease. **Objective**: To describe and compare the characteristics of mild, young and early COPD in a large sample of COPD from primary and secondary care. **Methods**: Pooled analysis of individual data from four multicenter observational studies of patients with stable COPD (≥40 years, FEV_1_/FVC < 0.7, smoking ≥ 10 pack-years). Mild COPD was defined as FEV_1_% ≥ 65%; young COPD as <55 years; and early COPD as <55 years and smoking ≤ 20 pack-years. The relationship between FEV_1_(%), age and pack-years was analyzed with linear regression equations. **Results**: We included 5468 patients. Their mean age was 67 (SD: 9.6) years, and 85% were male. A total of 1158 (21.2%) patients had mild COPD; 636 (11.6%) had young COPD and 191 (3.5%) early COPD. The three groups shared common characteristics: they were more frequently female, younger and with less tobacco exposure compared with the remaining patients. Early COPD had fewer comorbidities and fewer COPD admissions, but no significant differences were found in ambulatory exacerbations. In linear regression analysis, the decline in FEV_1_(%) was more pronounced for the first 20 pack-years for all age groups and was even more important in younger patients. **Conclusions**: Mild, young and early COPD patients were more frequently women. The steepest decline in FEV_1_(%) was observed in individuals <55 years and smoking between 10 and 20 pack-years (early COPD), which highlights the importance of an early detection and implementation of preventive and therapeutic measures.

## 1. Introduction

Chronic obstructive pulmonary disease (COPD) is a highly prevalent disease and one of the leading causes of death [1]. Epidemiological studies have estimated a prevalence of COPD of 11.8% in adults in Spain [2] and 13% worldwide [3], with a high rate of underdiagnosis, especially in women and younger adults [4]. The natural history of COPD is not yet fully understood and can be very variable; although smoking is still a major culprit [5], other genetic, environmental and developmental factors may exert their effects during the growing years by reducing the maximally attained forced expiratory volume in one second (FEV_1_) or accelerating FEV_1_ decline in adult life or both, thus increasing the risk of COPD [6].

The identification of young individuals at risk of developing COPD or at early stages of the disease could help to implement smoking cessation strategies and tackle symptom and exacerbation management and, hence, improve prognosis [7,8,9].

The concept of early disease is generally used to refer to disease of recent onset; however, in COPD, this concept is usually reserved for individuals who present with the disease at a young age irrespective of the time elapsed since the initiation of symptoms [10,11,12,13,14,15]. On the other hand, mild COPD is defined by a relatively preserved FEV_1_, irrespective of the age of the patient. As a consequence, the terms of mild and early COPD are sometimes confused with COPD in young subjects, or “young COPD” [16,17].

During the last years, operational definitions of early COPD have been proposed [18,19] that usually include young age (relative to the middle age of patients with COPD), airflow limitation and a significant smoking burden. In fact, the main difference between these proposed definitions of early COPD and the definition of COPD in general is the age range, but they do not take into account the time elapsed since the beginning of symptoms or the initiation of the causative exposure, which is predominantly smoking in most developed countries.

The objective of the present study was to describe and compare the characteristics of mild, young and early COPD on a large sample of Spanish COPD patients from primary and secondary care.

## 2. Materials and Methods

### 2.1. Design of the Study

This was a pooled analysis of individual-level data from 4 multicenter, cross-sectional, observational studies [19,20,21,22]. In summary, all studies included COPD patients recruited while in a stable state in both primary care and pneumology outpatient clinics. All individuals were ≥40 years old, smokers or former smokers of at least 10 pack-years with a spirometrically confirmed diagnosis of COPD defined by a post-bronchodilator [FEV_1_]/forced vital capacity [FVC] ≤ 0.7 and FEV_1_(%) of 80% or less.

### 2.2. Variables

Sociodemographic, clinical and functional data, as well as the number of exacerbations and hospital admissions due to COPD in the previous year, were collected. Dyspnea was assessed using the modified Medical Research Council (mMRC) dyspnea scale [23], which is a questionnaire that consists of five statements about perceived breathlessness: grade 1, “I only get breathless with strenuous exercise”; grade 2, “I get short of breath when hurrying on the level or up a slight hill”; grade 3, “I walk slower than people of the same age on the level because of breathlessness or have to stop for breath when walking at my own pace on the level”; grade 4, “I stop for breath after walking 100 yards or after a few minutes on the level”; and grade 5, “I am too breathless to leave the house”. The impact of the disease was assessed using the COPD assessment test (CAT) [24], which is a validated, short, self-administered questionnaire that measures the impact of the disease in patients with 8 questions; the score ranges from 0 to 40, with 40 being the worst possible health state and 0 the best. The BODEx (body mass index, airway obstruction, dyspnea and exacerbations) index was also included [25]; this is a severity score with prognostic value for exacerbations and mortality, with a score range from 0 to 9 units, with 9 being the worst prognosis. Self-reported comorbid conditions were registered according to the Charlson comorbidity index [26]; in general, absence of comorbidity is considered to be 0–1 point, low comorbidity 2 points and high comorbidity 3 or more points.

### 2.3. Definitions

The inclusion criteria of the selected studies included an age of at least 40 years and a post-bronchodilator FEV_1_(%) of 80% or less, and thus, mild COPD was arbitrarily defined as an FEV_1_(%) above 65% predicted, and young COPD was defined as the group of patients younger than 55 years old. We defined low smoking intensity as having an exposure between 10 to 20 pack-years, while >20 pack-years was considered high-intensity exposure. Therefore, early COPD was defined as an age younger than 55 years and smoking exposure of less than 20 pack-years. Although we are aware that in some patients COPD may start very early in life [27], with this definition we wanted to identify individuals who develop COPD at earlier stages of the disease compared to the remaining population. In order to further analyze the independent impact of age and smoking on the severity of airway limitation, patients were divided into four groups: (1) young low-intensity smokers (YLS), also defined as early COPD (<55 years old and ≤20 pack-years); (2) young high-intensity smokers (YHS) (<55 years old with >20 pack-years); (3) old low-intensity smokers (OLS) (≥55 years with ≤20 pack-years) and (4) old high-intensity smokers (OHS) (≥55 years old with >20 pack-years). According to current guidelines, severe COPD is defined by an FEV_1_(%) 30–50% and very severe COPD by an FEV_1_(%) < 30% [28].

### 2.4. Statistical Analysis

The categorical variables were described with absolute frequencies and percentages. The description of quantitative variables was carried out using the mean and standard deviation (SD). The Kolmogorov–Smirnov test was used to evaluate the normality of the distributions. Sociodemographic and clinical characteristics were compared according to the age of the participants, tobacco consumption (pack-years) and lung function. In the case of quantitative variables, the Mann–Whitney U or Kruskal–Wallis tests, with Bonferroni correction for multiple comparisons, were carried out. The Chi-squared test (Fisher test for frequencies <5) was used for the comparison of categorical variables. Linear regression equations were performed to analyze the relationship between FEV_1_(%), age and pack-years. For all tests, *p* values <0.05 were considered statistically significant. The R Studio statistical package (V4.3.3) was used for the analyses.

## 3. Results

### 3.1. Population

We screened 7520 patients with COPD, and among these, 5468 (72.7%) had complete and valid data on age, pulmonary function and smoking habit and were included in this analysis. The mean age was 67 (SD: 9.6) years, and 85% were male with a mean smoking consumption of 43.9 pack-years (25.4) (Table 1).

### 3.2. Mild COPD

A total of 1158 (21.2%) patients had mild COPD. Compared to more severe patients, those with mild COPD were younger (65.4 (10.1) vs. 67.4 (SD: 9.4) years, *p* < 0.001), more frequently female (24.4% vs. 12%, *p* < 0.001) and had lower tobacco exposure (39.1 (23.2) vs. 45.2 (25.8) pack-years; *p* < 0.001). As expected, they also had fewer symptoms, with lower mMRC and CAT scores and significantly less frequent exacerbations and hospital admissions. There were no differences in comorbidities recorded with the Charlson index (Table 1).

### 3.3. Young COPD

The group of young COPD consisted of 636 (11.6%) individuals younger than 55 years old. They were more frequently female (31% vs. 12.5%, *p* < 0.001) and with lower tobacco exposure (32.4 (19.0) vs. 45.5 (25.7) pack-years, *p* < 0.001). They also had fewer symptoms, with a lower CAT score. Differences in BODEx and FEV_1_, although statistically significant, were of small magnitude. No differences were found regarding the history and type of exacerbations (Table 2).

### 3.4. Early COPD

The group defined as early COPD consisted of 191 patients (3.5%), who were more frequently women (42.4% vs. 13.6%, *p* < 0.001) and had fewer comorbidities measured by the Charlson index than the remaining patients (1.5 (0.8) vs. 2.1 (1.1), *p* < 0.001). The FEV_1_(%) was better preserved in the early COPD group (58.4% (18.3%) vs. 50.4% (16.9%), *p* < 0.001), and they had fewer COPD admissions (0.5 (1.1) vs. 0.8 (1.4), *p* < 0.001), but no significant differences were found in the ambulatory COPD exacerbations (1.5 (1.9) vs. 1.6 (1.9), *p* = 0.418) (Table 3).

### 3.5. Comparison Between Groups Classified According to Age and Smoking Intensity

As previously described, younger patients were more frequently female, but even among younger patients, those with lower smoking exposure were significantly more frequently female compared with the high-intensity smoker young COPD patients (42.4% vs. 26.1%; *p* < 0.001).

There was a clear effect of age on comorbidities, with both groups of elderly patients (OLS and OHS) having a higher Charlson index compared to younger patients. However, a significant effect of smoking on comorbidities was only observed in young patients (1.5 (0.8) in YLS versus 1.7 (0.9) in YHS; *p* < 0.001).

Regarding lung function, not surprisingly, OHS had the worse FEV_1_(%) (49.7%) and YLS the best FEV_1_(%) (58.4%); but interestingly, FEV_1_(%) values did not significantly differ between YHS and OLS (52.8% vs. 52.6%). Nonetheless, dyspnea was significantly worse in OLS compared with YHS (mMRC of 1.5 (0.9) vs. 1.3 (0.9); *p* < 0.001). Dyspnea did not differ according to smoking but only according to age, and the same was true for symptoms measured by the CAT scores.

We observed no significant differences in the frequency of exacerbations among the four groups, but there was a combined effect of age and smoking on severe exacerbations, with only YLS having significantly less frequent severe exacerbations compared to the two groups of older patients (Table 4).

**Figure 1:**
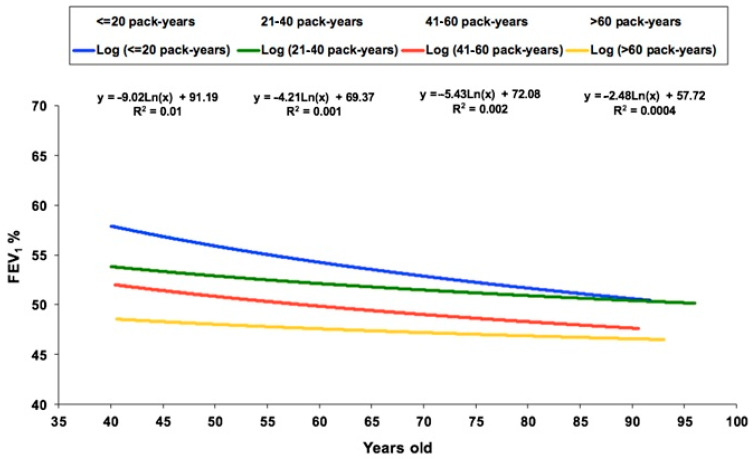
Linear regression equations of FEV_1_(%) and age according to the different groups of smoking exposure.

When plotting FEV_1_(%) versus age for the participants divided into four groups according to smoking consumption, we observed that at a younger age there were important differences in FEV_1_(%) according to the pack-years of smoking, but these differences progressively reduced with increasing age. In particular, there was a clear reduction in FEV_1_(%) with age in smokers of less than 20 pack-years, whereas the line was almost flat for smokers of more than 60 pack-years (Figure 1).

**Figure 2:**
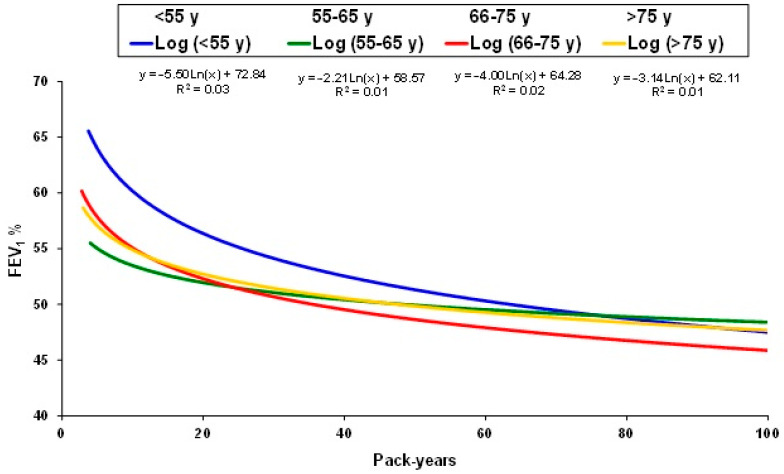
Linear regression equations of FEV_1_(%) and smoking exposure according to the different age groups.

When plotting FEV_1_(%) versus pack-years by age subgroups, we observed that for all age groups the slope of decline in FEV_1_(%) was more pronounced for the first 20 pack-years, and again, this was more important in younger patients. Furthermore, this effect was more pronounced in individuals younger than 55 years old with less than 20 pack-years (early COPD) (Figure 2).

## 4. Discussion

In our large population of patients with COPD, around one fifth had mild COPD, and these patients were younger, more frequently female, with lower tobacco exposure and less symptomatic. Similarly, young COPD patients were also more frequently female, with lower tobacco exposure, less symptomatic and with a lower CAT score. On the other hand, patients defined as early COPD had a higher proportion of women and fewer comorbidities, their FEV_1_(%) was better preserved and they had fewer COPD admissions. However, differences in the frequency of ambulatory COPD exacerbations were not significantly different among the previous three groups of patients and the remaining patients with COPD. When analyzing the effect of age and tobacco exposure, we observed that the presence of comorbidities and symptoms were mainly influenced by age more than by smoking habits. In contrast, no differences were observed in the frequency of ambulatory exacerbations among the four different groups according to age and smoking exposure, while severe exacerbations were more frequent in elderly patients with high tobacco exposure.

Our large database provided an ideal opportunity to analyze different aspects of patients with COPD at early stages of the disease. First, we studied the characteristics of patients considered mild according to their level of impairment in FEV_1_(%). The most accepted definition of mild COPD includes patients with a post-bronchodilator FEV_1_/FVC < 0.7 and a FEV_1_(%) > 80% predicted [28,29,30]. However, older definitions of mild disease, such as that used in the Lung Health Study by Anthonisen et al. [31], included patients with a FEV_1_(%) between 55–90%. Likewise, in other studies, the term “mild–moderate” was used for patients with a FEV_1_(%) ≥ 50% or GOLD II [32,33,34]. Since the population included in our study had a FEV_1_(%) below 80% as an inclusion criterion, we arbitrarily set the cut-off point for “mild COPD” at FEV_1_(%) ≥ 65%. Consequently, our results in mild COPD may not be completely comparable to other series defined by FEV_1_(%) > 80%. In any case, it may be useful to compare the characteristics of patients who have a less affected pulmonary function with the remaining “more severe” COPD patients. If we compare our group of mild COPD with other studies [8,32], we observe that they share common characteristics, such as having a higher proportion of women and less tobacco exposure. However, our mild patients had fewer symptoms than the groups classified as GOLD II, probably explained by the more preserved FEV_1_ of our cohort (65–80%). Conversely, our cohort of mild COPD had a higher exacerbation rate compared with the GOLD I-II patients described in the systematic review of Maltais et al. [8], which could be explained by the older mean age of our patients.

Several studies define young COPD as patients younger than 50 years old [12,35,36,37]; however, we used the threshold of 55 years because all our population was >40 years, and there were very few patients between 40 and 50 years of age. Nevertheless, our results were similar to those of other series [35,36,37], showing that young COPD patients were more frequently female, with less tobacco exposure and with a more preserved FEV_1_ than older patients. Interestingly, we found that the relationship between smoking and FEV_1_(%) showed the steepest slope in FEV_1_(%) in subjects <55 years compared with the other age groups. This is in agreement with previous studies that suggest that COPD may progress more rapidly in younger patients with a significant smoking history [10]. If we compare our data with a recent study by Tan et al. [37], who defined young COPD as patients between 20–50 years old, we find that our patients were more symptomatic. This may be explained, at least in part, because in their series 75% were never smokers, and the mean average of tobacco consumption among the smokers was only 5 pack-years. Moreover, their young COPD patients had a significant FEV_1_(%) reversibility and preserved diffusion capacity, which suggests that some of these patients might be affected by asthma instead. To avoid the misdiagnoses of young COPD in subjects with asthma, a significant smoking history or other exposures should be required.

Early COPD has gained increasing attention during recent years, although the lack of a universally accepted definition hinders the comparison of studies. An operational definition of early COPD has been proposed [12] as individuals younger than 50 years with 10 or more pack-years of smoking and one or more of the following abnormalities: (1) early airflow limitation, (2) compatible computerized tomography abnormalities and/or (3) a rapid decline in FEV_1_ (>60 mL/year). However, other definitions use only age, smoking and airflow limitation [10]. For example, Çolak et al. [38] and Cosío et al. [35] defined early COPD as individuals under 50 years of age with obstructive spirometry and a 10 pack-year or greater tobacco consumption.

As previously indicated, we used a threshold of 55 years in our series, and, since all our patients had at least 10 pack-years of tobacco exposure, we limited smoking to 20 pack-years in the definition of the early COPD group. This limit was established because some smokers may have already accumulated a substantial number of pack-years at a young age and, therefore, should not be considered to be initiating the damage to their lungs. Therefore, we defined early COPD as <55 years old and ≤20 pack-years, because these characteristics might select individuals closer to a real initial stage of the disease.

With our definition, we found that the patients classified as early COPD were more frequently women, with fewer comorbidities and with a better preserved FEV_1_(%) compared with the remaining COPD patients. Nonetheless, no significant differences were found in the frequency of ambulatory COPD exacerbations, which could be explained, in part, by a possible diagnostic bias, because in our early COPD cohort, patients were recruited after seeking care for symptoms or exacerbations; therefore, early COPD patients with lower symptom levels or without exacerbations may have remained undiagnosed.

When comparing our cohort of early COPD with other series [35,38], due to our definition, our patients were older and had less tobacco exposure. Despite that, the FEV_1_(%) of our patients was lower than that described by Çolak et al. [38], and, in addition, our population was more symptomatic and with a higher CAT score (16.6 vs. 12.5) than the early COPD patients described by Cosío et al. [35]. The higher severity of our population of early COPD (despite a lower smoking consumption) may be explained not only by the older mean age but also because our population consisted of previously diagnosed COPD patients followed in outpatient pulmonology clinics, while the previously described series derived from automatically generated lists of smokers from primary care [35] and from a population-based cohort [38]. Moreover, other characteristics, such as the proportion of asthma–COPD overlap patients in the different groups or the adherence to inhaled therapy, were not registered in the original studies and therefore could not be assessed in the current pooled analysis [39]. Our early COPD patients are surely in more initial stages than the rest of the group, but their mean FEV_1_(%) of 54.8% suggests that they are already in a moderately advanced stage of the disease. To truly identify patients in early stages of COPD, we should improve the early diagnosis of COPD, probably through case detection strategies [40,41,42,43].

These findings are relevant, because mild, young and early COPD patients presented a similar rate of moderate exacerbations compared to more severe or elderly patients, and exacerbations are associated with a more rapid decline in lung function, worse quality of life and decreased exercise capacity [44,45,46]. Moreover, we observed a steeper decline in FEV_1_(%) in individuals under 55 years of age with less than 20 pack-years, which highlights the importance of an early detection and implementation of preventive and therapeutic measures.

Our definitions of mild, young and early COPD are not mutually exclusive, and, therefore, patients included in these categories share several common characteristics. The most important of these shared characteristics is the higher prevalence of females compared with the remaining patients, and they are also less symptomatic, with a better CAT score and less frequent severe exacerbations. The higher frequency of women in early stages of the disease has also been observed in other studies [47,48] and could be due to a greater susceptibility of women to smoking [49]. Furthermore, the rate of underdiagnoses of COPD is greater in women [4,50], in part due to differences in the attitudes of physicians towards women with respiratory symptoms, with the risk of being mislabeled as asthmatics [51]. Therefore, accurately identifying this group of patients should be of particular interest for early diagnosis and treatment.

A combined effect of age and smoking was observed with the Charlson comorbidity index. Increasing age has been correlated with an increase in comorbidities [52,53], and, similarly, a higher smoking load has also been associated with more comorbidities. In contrast, we found no significant differences in the frequency of ambulatory exacerbations according to age and smoking exposure, but age had a significant impact on severe exacerbations, in agreement with the widely recognized effect of aging as a risk factor for admissions [54].

The main limitation of our analysis is that our data were cross-sectional, and therefore we could not analyze the natural course of the different forms of COPD. Furthermore, due to the inclusion criteria of the selected studies, our definitions of mild, young and early COPD may differ from other definitions, which could make direct comparisons between studies more challenging. However, we believe that the large study sample from all areas from Spain and from primary and secondary care provides a unique opportunity to characterize these “mild” patients.

## 5. Conclusions

The definition of early COPD should include variables that identify patients in a stage close to the onset of the disease. The concepts of mild or young patients may not be accurate enough to define this group. Among the young and mild patients, there are more women than in the rest of the groups, a characteristic that is shared with our definition of early COPD. The steepest decline in FEV_1_(%) was observed in individuals under 55 years of age with less than 20 pack-years of smoking, which highlights the importance of an early detection and implementation of preventive and therapeutic measures. These results must be confirmed in longitudinal studies.

## Figures and Tables

**Table 1:** Baseline characteristics of the population and comparisons between mild COPD and the remaining patients with COPD.

Parameter	Subjects (N = 5468)	Mild COPD * N = 1160	COPD with FEV_1_ < 65% N = 4308	*p*-Value ^a^
Age (years)	67 (9.6)	65.4 (10.1)	67.4 (9.4)	<0.001
Pack-years	43.9 (25.4)	39.1 (23.1)	45.2 (25.7)	<0.001
Sex, male (%)	4669 (85.4)	876 (75.5)	3788 (88.0)	<0.001
BMI (kg/m^2^)	27.6 (4.6)	27.7 (4.6)	27.5 (4.5)	0.249
Charlson index	2 (1.1)	2.01 (1.0)	2.04 (1.0)	0.232
mMRC	1.50 (0.9)	1.22 (0.6)	1.58 (0.8)	<0.001
FEV_1_ (mL)	1519 (583)	2213 (508)	1340 (452)	<0.001
FEV_1_(%)	50.6 (17)	75 (8.1)	44.1 (12.1)	<0.001
FEV_1_/FVC	53.6 (11.1)	61.9 (6.7)	51.3 (10.9)	<0.001
Ambulatory exacerbations	1.6 (1.9)	1.59 (1.6)	1.65 (1.9)	0.943
Admissions	0.8 (1.4)	0.54 (1.0)	0.90 (1.5)	<0.001
Total exacerbations	2.5 (2.7)	2.12 (2.1)	2.55 (2.8)	<0.001
CAT	18.3 (8.6)	15.9 (8.7)	18.9 (8.4)	<0.001
BODEx index	2.6 (1.7)	0.8 (0.9)	3.1 (1.5)	<0.001

Footnote: * Mild COPD is defined as FEV_1_(%) ≥ 65%. Data are presented as mean (SD) unless otherwise specified. Kruskal–Wallis test ^a^. Abbreviations: BMI: body mass index, mMRC, modified Medical Research Council dyspnea score, FEV_1_: forced expiratory volume in one second, CAT, COPD assessment test; BODEx, body mass index, airway obstruction, dyspnea, exacerbation.

**Table 2:** Comparisons of characteristics between mild COPD and the remaining patients with COPD.

Parameter	Age < 55 Years Old N = 636	Age ≥ 55 Years Old N = 4827	*p*-Value ^a^
Age (years)	50.3 (3.5)	69.2 (7.9)	<0.001
Pack-years	32.4 (19.0)	45.5 (25.7)	<0.001
Sex, male (%)	439 (69.0)	4225 (87.5)	<0.001
BMI (kg/m^2^)	27 (5.3)	27.6 (4.4)	<0.001
Charlson index	1.64 (0.8)	2.08 (1.0)	<0.001
mMRC	1.32 (0.7)	1.59 (0.7)	<0.001
FEV_1_ (mL)	1797 (644)	1482 (564)	<0.001
FEV_1_(%)	54.5 (17.9)	50.1 (16.8)	<0.001
FEV_1_/FVC	55.1 (10.9)	53.4 (11.1)	<0.001
Ambulatory exacerbations	1.65 (1.9)	1.59 (1.6)	0.557
Admissions	0.69 (1.3)	0.84 (1.4)	0.021
Total exacerbations	2.27 (2.5)	2.49 (2.7)	0.063
CAT	16.6 (8.6)	18.5 (8.6)	<0.001
BODEx index	2.2 (1.7)	2.6 (1.6)	<0.001

Footnote: Data are presented as mean (SD) unless otherwise specified. Kruskal–Wallis test ^a^. Abbreviations: BMI: body mass index, mMRC, modified Medical Research Council dyspnea score, FEV_1_: forced expiratory volume in one second, CAT, COPD assessment test; BODEx, body mass index, airway obstruction, dyspnea, exacerbation.

**Table 3:** Comparisons of characteristics between early COPD and the remaining patients with COPD.

Parameter	Early COPD * (N = 191)	No Early COPD (N = 5272)	*p*-Value ^a^
Age (years)	49.7 (3.9)	67.6 (9.2)	<0.001
Pack-years	15.3 (3.7)	45 (21.2)	<0.001
Sex, male (%)	110 (57.6)	4554 (86.4)	< 0.001
BMI (kg/m^2^)	26.6 (4.7)	27.6 (4.5)	<0.001
Charlson index	1.5 (0.8)	2.1 (1.1)	<0.001
mMRC	1.3 (0.9)	1.5 (0.9)	<0.001
FEV_1_ (mL)	1848 (641)	1508 (577)	<0.001
FEV_1_(%)	58.4 (18.3)	50.4 (16.9)	<0.001
FEV_1_/FVC	56.7 (11.1)	53.5 (11)	<0.001
Ambulatory exacerbations	1.5 (1.9)	1.6 (1.9)	0.418
Admissions	0.5 (1.1)	0.8 (1.4)	<0.001
Total exacerbations	2 (2.4)	2.5 (2.8)	0.009
CAT	16.6 (8.3)	18.3 (8.7)	0.026
BODEx index	1.9 (1.6)	2.6 (1.7)	<0.001

Footnote: * Early COPD is defined as age < 55 years and smoking exposure ≤ 20 pack-years. Data are presented as mean (SD) unless otherwise specified. ^a^ Mann–Whitney, o Chi-square test. Abbreviations: BMI: body mass index, mMRC, modified Medical Research Council dyspnea score, FEV_1_: forced expiratory volume in one second, CAT, COPD assessment test, BODEx, body mass index, airway obstruction, dyspnea, exacerbation.

**Table 4:** Comparison of demographic, functional and clinical characteristics of patients according to age and accumulated smoking consumption.

	Young Patients		Old Patients		
Parameter	Young and Low Smoking (Early COPD) (N = 191)	Young and High Smoking (N = 445)	Old and Low Smoking (N = 740)	Old and High Smoking (N = 4092)	*p*-Value ^a^
Age (years)	49.7 (3.9)	50.6 (3.3)	68.1 (8.1)	69.3 (7.9)	<0.001 ^cdfg^
Pack-years	15.3 (3.7)	39.8 (18.2)	15.5 (3.8)	50.8 (24.2)	<0.001 ^bdfg^
Sex, male (%)	110 (57.6)	329 (73.9)	567 (76.6)	3658 (89.5)	<0.001 ^bcd^
BMI (kg/m^2^)	26.6 (4.7)	27.2 (5.6)	27.6 (4.4)	27.6 (4.5)	<0.001 ^cd^
Charlson index	1.5 (0.8)	1.7 (0.9)	2.1 (1.1)	2.1 (1.1)	<0.001 ^cdef^
mMRC	1.3 (0.9)	1.3 (0.9)	1.5 (0.9)	1.5 (0.9)	<0.001 ^cdef^
FEV_1_ (mL)	1848 (640)	1776 (645)	1520 (562)	1476 (564)	<0.001 ^cde^
FEV_1_(%)	58.4 (18.3)	52.8 (17.1)	52.6 (17.4)	49.7 (16.7)	<0.001 ^bcdfg^
FEV_1_/FVC	56.7 (11.1)	54.4 (10.7)	54.9 (11)	53.1 (11)	<0.001 ^bdg^
Ambulatory exacerbations	1.5 (1.9)	1.6 (1.6)	1.7 (1.9)	1.6 (1.9)	0.873
Admissions	0.5 (1.1)	0.8 (1.5)	0.9 (1.7)	0.8 (1.4)	<0.001 ^cd^
Total exacerbations	2 (2.4)	2.4 (2.6)	2.6 (3.1)	2.5 (2.7)	0.023 ^c^
CAT	16.6 (8.3)	16.6 (8.8)	18.2 (8.8)	18.5 (8.6)	0.001 ^f^
BODEx index	1.9 (1.6)	2.3 (1.8)	2.4 (1.7)	2.7 (1.7)	<0.001 ^cdfg^

Footnote: Data are presented as mean (SD) unless otherwise specified. Kruskal–Wallis test ^a^. Abbreviations: BMI: body mass index, mMRC, modified Medical Research Council dyspnea score, FEV_1_: forced expiratory volume in one second, CAT, COPD assessment test; BODEx, body mass index, airway obstruction, dyspnea, exacerbation. (YLS vs. YHS) ^b^, (YLS vs. OLS) ^c^, (YLS vs. OHS) ^d^, (YHS vs. OLS) ^e^, (YHS vs. OHS) ^f^, (OLS vs. OHS) ^g^.

## Data Availability

The data presented in this study are available on request from the corresponding author.
